# Perioperative immune checkpoint inhibitors plus chemotherapy versus chemotherapy alone in resectable gastric and gastroesophageal junction adenocarcinoma: a systematic review and meta-analysis

**DOI:** 10.3126/nje.v16i1.92321

**Published:** 2026-04-02

**Authors:** Hyder Mohammad Waseem Din, Yamini Saraswathi Gurram, Aimen Mansur, Mohammad Maroof Shahid, Tayyaba Binte Saleem, Karoona Kumari, Beesham Kumar, Pershan Kumar, Maira Shahid, Mahmud Seyi Abdurrahman, Muneeb Khawar

**Affiliations:** 1 Altnagelvin Area Hospital, Londonderry, United Kingdom; 2 Dr. Pinnamaneni Siddhartha Institute of Medical Sciences & Research Foundation, Vijayawada, India; 3 King Edward Medical University, Lahore, Pakistan; 4 Services Institute of Medical Sciences, Lahore, Pakistan; 5 Memorial Healthcare System, Hollywood, Florida, United States; 6 Liaquat University of Medical & Health Sciences, Jamshoro, Pakistan; 7 Jinnah Medical & Dental College, Karachi, Pakistan; 8 Faisalabad Medical University, Faisalabad, Pakistan; 9 Richmond Gabriel University, Kingstown, St. Vincent and the Grenadines

**Keywords:** Immune checkpoint inhibitors, Gastric cancer, Perioperative chemotherapy, Meta-analysis

## Abstract

**Background:**

Gastric and gastroesophageal junction (GEJ) cancer is not responsive to surgery and perioperative chemotherapy. Immune checkpoint inhibitors (ICIs) have been effective in advanced disease. This meta-analysis assesses the benefits of the addition of ICIs to perioperative chemotherapy in case of resectable gastric/GEJ cancer.

**Methods:**

PubMed, Embase, and ScienceDirect were identified until February 2026. Randomized controlled trials with comparison of perioperative ICI with chemotherapy against chemotherapy were included. A random-effects model was used to. Risk ratios was calculated for all the dichotomous outcomes. I^2^ was used to assess the heterogeneity.

**Results:**

Four trials (2,125 patients) were included. ICI plus chemotherapy, however, had significant positive effects on pathological complete response (RR = 2.87; 95% CI: 1.66-4.99; p = 0.018) and complete tumour regression (RR = 2.74; 95% CI: 1.24-6.04; p = 0.006). No significant different was found for R0 resection rates and surgery completion between the two groups. Similarly, no significant different was found for adverse events, surgical morbidity, or deaths. However, immune-related adverse events (RR = 3.18; 95% CI: 2.48–4.08) and treatment discontinuation (RR = 1.31; 95% CI: 1.11–1.54) were significantly higher in ICI plus chemotherapy group.

**Conclusion:**

The inclusion of ICIs in perioperative chemotherapy enhances pathological response and does not pose more surgical risk. Although adverse events were high in the ICI plus chemotherapy group so large scale studies are needed to validate these findings.

## Introduction

Gastric cancer is a major health concern burden in the world, as it is the fifth most common malignancy, and the fourth most important cause of cancer death in the entire world [1]. The estimates provided by GLOBOCAN 2022 show that around 968,000 new cases and 660,000 deaths could be attributed to gastric cancer worldwide [2]. The prognosis of patients with locally advanced resectable gastric and gastroesophageal junction (GEJ) adenocarcinoma is still poor with five-year survival rates of less than 50% [3]. Perioperative chemotherapy surgery has been instituted as a standard of care, when dealing with resectable gastric and GEJ adenocarcinoma in the West. The MAGIC trial, which was the first to report this, showed that overall survival in perioperative epirubicin, cisplatin, and fluorouracil (ECF) was significantly better than that using surgery alone, with five-year rates of 36% versus 23% [4]. Then the FLOT4 trial demonstrated that perioperative use of fluorouracil, leucovorin, oxaliplatin and docetaxel (FLOT) was better than the previously used ECF/ECX with a median overall survival of 50 months chemotherapy [3]. In spite of these innovations, a significantly large percentage of patients recur their disease and succumb to their illness, which highlights the necessity of new therapeutic approaches to enhance the results even more [5].

Anti-PD-1 and anti-PD-L1 immunomodulators have revolutionized the therapy of advanced gastric cancer. CheckMate 649 trial was used to define nivolumab with chemotherapy as an initial first-line treatment in the management of HER2-negative advanced gastric and GEJ adenocarcinoma [6]. It has been suggested by the success of ICIs in the metastatic environment that multiple phase II studies and phase III have been conducted to evaluate the use of ICIs in the perioperative therapy of locally advanced resectable disease. The KEYNOTE-585 trial compared preoperative pembrolizumab with chemotherapy and was able to show a greatly enhanced rate of pathological complete response (pCR) but failed to be statistically significant in terms of event-free survival (EFS) [7]. According to the MATTERHORN trial, perioperative durvalumab plus FLOT was found to be significantly better than FLOT in terms of pCR as well as EFS [8]. Smaller trials like DANTE trial or the study by Yuan et al. have also provided more evidence to support the use of this approach [9]. Findings in these trials have however been inconsistent especially on long term survival outcomes and their safety is an ongoing study issue.

This systematic review and meta-analysis will fill that gap by conducting a synthesis of the evidence provided by all available direct-comparison trials to assess the relative effectiveness and safety of adjunctions of ICIs to perioperative chemotherapy in this group of patients.

## Methodology

The systematic review and meta-analysis study were carried out in accordance with Preferred Reporting Items of Systematic Reviews and Meta-Analyses (PRISMA) 2020 guidelines [11]. The protocol of the study was enrolled in PROSPERO (CRD420261352704).

### Search Strategy

The systematic electronic search of the literature was conducted in PubMed, Embase, Cochrane Central Database of Controlled Trials (CENTRAL), and Web of Science since the database inception until February 2026. The search strategy involved the Medical Subject Headings (MeSH) terms and the free-text words, such as: (immune checkpoint inhibitor' OR immunotherapy' OR nivolumab' OR pembrolizumab' OR sintilimab' OR atezolizumab' OR duralvalumab) AND (gastric cancer' OR gastroesophageal junction' OR stomach neoplasm) AND (perioperative' OR neoadjuvant' OR preoperative'. Manual screening was done to add more records to the reference lists of included studies.

### 2.2 Eligibility Criteria

The studies were added when they satisfied the following criteria: (1) randomized controlled trials with comparison between perioperative ICI and chemotherapy and chemotherapy alone; (2) participated in the studies were adult patients (age 18 or older) with resectable or locally advanced gastric or GEJ adenocarcinoma; (3) at least one efficacy or safety outcome. The non-randomized studies, single-arm trials, case reports, editorials, and narrative reviews were excluded.

### Study Selection

Titles and abstracts were screened by two reviewers and then there was a full-text review of potentially relevant articles against the preset inclusion criteria. Any disagreements at each of the stages were solved by the third reviewer.

### Data Extraction

Data extraction was done independently by two reviewers who used a standardised extraction form. The variables included first author name, year of publication, trial name, study design, ICI drug name and dosing regimen, chemotherapy backbone, sample size in each group, follow-up duration. Furthermore, the baseline characteristics like the demographic variables of the participants including age, sex, tumour location, clinical stage, PD-L1 combined positive score, microsatellite instability status, Lauren classification, and ECOG performance status were also extracted. For dichotomous outcomes, the number of events and total participants for each group was documented.

### Quality Appraisal

The risk of bias of each of the included trials was assessed using the Cochrane Risk of Bias 2 (RoB 2) tool [12], which evaluates five domains. Each domain was finally scored as low risk, some concerns, or high risk. Assessments were performed by two reviewers independently and any disagreements were solved by a third reviewer.

### Statistical Analysis

All the analyses were done with R version 4.5.1 of. For dichotomous outcomes, risk ratios (RR) with a 95% confidence interval (CI) was obtained using the Mantel-Hansel method. A random effects model was used for the analysis. Heterogeneity was examined by means of the I^2^ statistic and Cochran's Q test. A significance level of p < 0.05 was made. The publication bias was not assessed considering small number of studies in this meta-analysis.

## Results

### Study Selection

**Figure 1:**
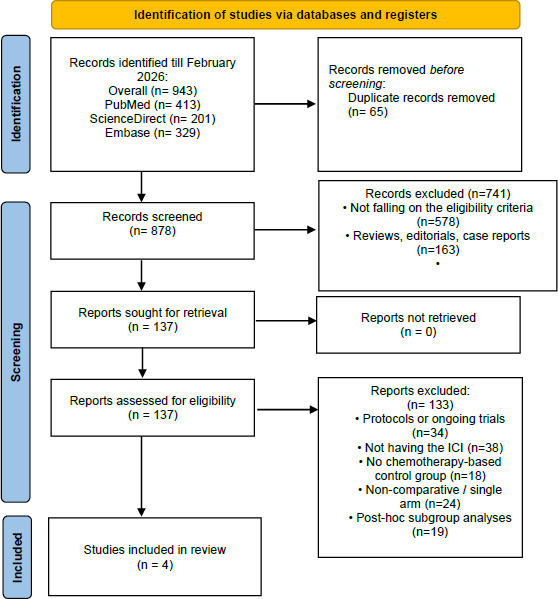
PRISMA flowchart

The PRISMA-flowchart (Figure 1) summarizes the study selection procedure. A detailed electronic literature search up to February 2026 using 3 different electronic databases yielded 725 records (PubMed: n = 413; Embase: n = 201; ScienceDirect: n = 329). After records were de-duplicated, 878 titles and abstracts were screened of which 741 were excluded. Full text reports of the other 137 records were then sought for retrieval and evaluated for eligibility. Of these 133 reports were excluded resulting in the inclusion of 4 [7-10] studies in the systematic review and meta-analysis.

### Characteristics of Included Studies

Four randomized controlled trials were included in this meta-analysis. Shitara et al. 2024 (phase 3 double-blind RCT, pembrolizumab plus cisplatin/capecitabine or 5-FU, n=804, median follow-up 47.7 months), Yuan et al. 2024 (phase 2 open-label RCT, toripalimab plus SOX or XELOX, n=108), Lorenzen et al. 2024 (phase II/III open-label RCT, atezolizumab plus FLOT, n=295), and Janjigian et al. 2025 (phase 3 double-blind RCT, durvalumab plus FLOT, n=948, median follow-up 31.5 months). The pooled sample comprised 2,155 participants (1,076 ICI+chemotherapy, 1,079 chemotherapy alone). Patient populations were largely comparable across trials with median age 58–64 years, predominantly male, and ECOG PS 0–1. Detailed baseline characteristics are presented in Table 1.

### Clinical Outcomes

**Figure 2.**
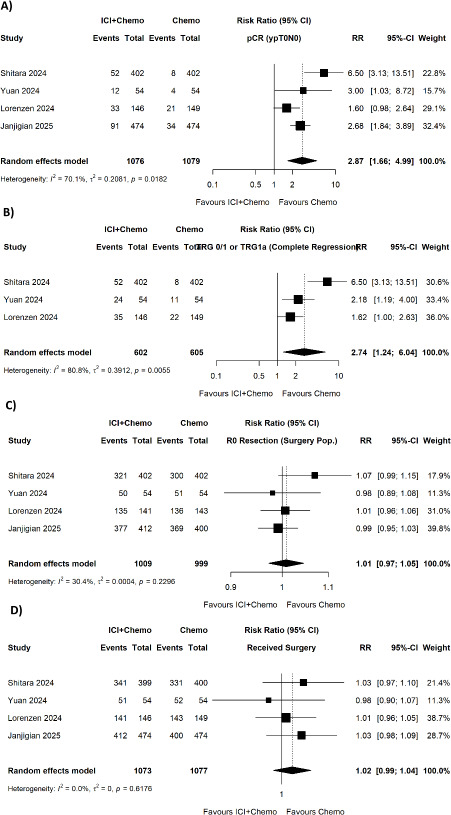
Forest plots of comparison of ICI + chemotherapy vs. chemotherapy only for (A) pathological complete response (pCR, ypT0N0), (B) complete regression (TRG 0/1 or RG 0/1), (C) R0 resection rate (surgery population), and D. proportion of the patients who received surgery.

ICI plus chemotherapy was significantly associated with a higher pathological complete response rate compared to chemotherapy alone (pooled RR = 2.87, 95% CI 1.66-4.99; p = 0.0182; I^2^ = 70.1%) (Fig. 2A) and a higher rate of complete tumour regression (pooled RR = 2.74, 95% CI 1.24-6.04; p = 0.0055; I^2^ = 80.8%) (Fig 2B). No significant difference between the use of suture and stitches in R0 resection rate (pooled RR = 1.01, 95% CI (0.97-1.05); p = 0.2296; I^2^ = 30.4%) (Fig. 2C) or surgery completion was observed (pooled RR = 1.02, 95% CI (0.99-1.04); p = 0.6176; I^2^ = 0.0%) (Fig. 2D).

**Figure 3.**
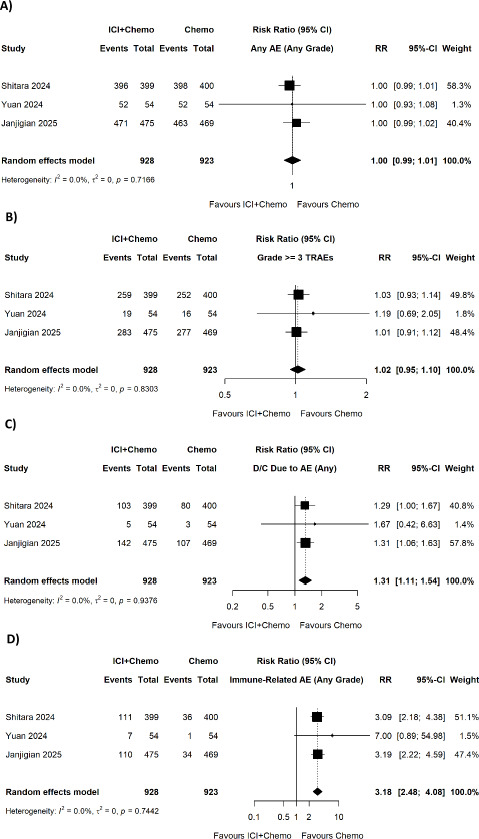
Forest plots of comparison of ICI + chemotherapy vs. chemotherapy only for (A) any adverse event (any grade), (B) grade ≥3 treatment-related adverse events, (C) discontinuation due to adverse events (any), and (D) immune-related adverse events (any grade).

No significant difference was found with regard to any-grade adverse events (pooled RR = 1.00, 95% CI 0.99-1.01, p = 0.7166, I^2^ = 0.0%) (Fig. 3A), treatment-related adverse events grade >=3 (pooled RR = 1.02, 95% CI 0.95-1.10, p = 0.8303, I^2^ = 0.0%) (Fig. 3B), surgical morbidity. However, ICI and chemotherapy was linked to significantly higher incidence of treatment discontinuation due to adverse event (pooled RR = 1.31, 95% CI 1.11-1.54; I^2^ = 0.0%) (Fig. 3C) and immune-related adverse events (pooled RR = 3.18, 95% CI 2.48-4.08; p < 0.001; I^2^ = 0.0%) (Fig. 3D).

**Figure 4.**
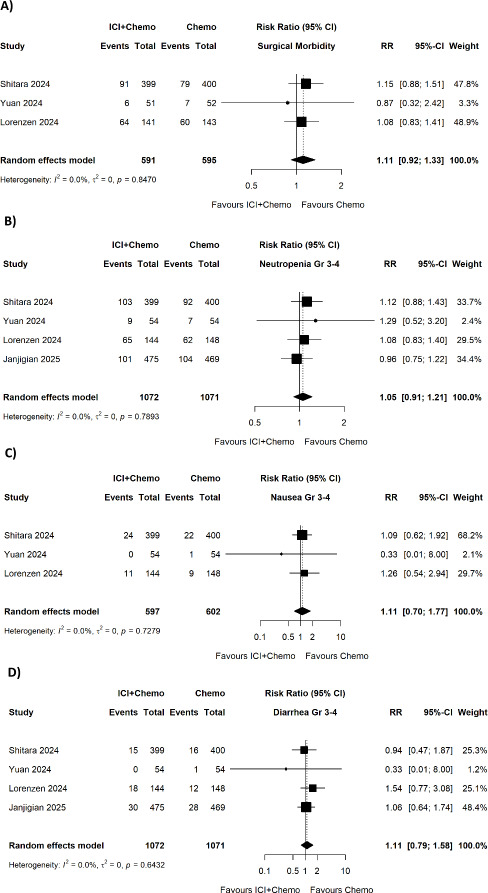
Forest plots showing the comparison of ICI plus chemotherapy versus chemotherapy alone for (A) surgical morbidity, (B) neutropenia grade 3–4, (C) nausea grade 3–4, and (D) diarrhea grade 3–4.

No significant difference was found in surgical morbidity (pooled RR = 1.11, 95% CI 0.92–1.33; p = 0.8470; I^2^ = 0.0%) (Fig. 4A), neutropenia grade 3–4 (pooled RR = 1.05, 95% CI 0.91–1.21; p = 0.7893; I^2^ = 0.0%) (Fig. 4B), nausea grade 3–4 (pooled RR = 1.11, 95% CI 0.70–1.77; p = 0.7279; I^2^ = 0.0%) (Fig. 4C), or diarrhea grade 3–4 (pooled RR = 1.11, 95% CI 0.79–1.58; p = 0.6432; I^2^ = 0.0%) (Fig. 4D).

### Quality assessment

**Figure 5.**
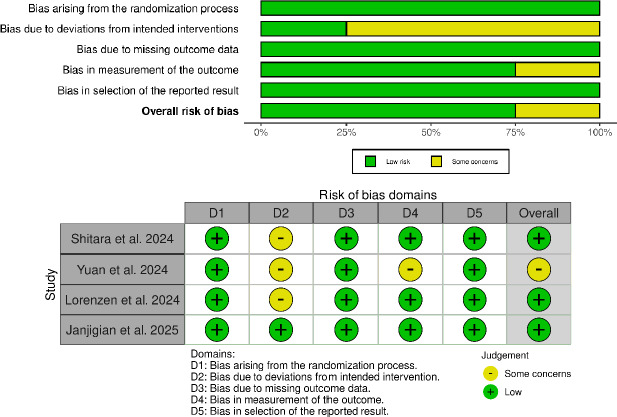
Summary and traffic light plot of risk of bias assessment using RoB 2.0 tool.

Risk of bias of the included studies was evaluated by using the RoB 2.0 tool (Figure 5). Shitara et al. 2024, Lorenzen et al. 2024 and Janjigian et al. 2025 were judged as having a low risk of bias on all domains. Yuan et al. 2024 was rated as having some concerns, mainly in Domain 2 with the existence of possible deviations from intended interventions in the perioperative setting. Consequently, the overall risk of bias was judged as low risk of bias in three studies, and some concerns in one study..

## Discussion

This meta-analysis of four randomized controlled trials is a comprehensive synthesis of the efficacy and safety of the use of immune checkpoint inhibitors in the perioperative setting with chemotherapy for resectable gastric and gastroesophageal junction adenocarcinoma. Our principal findings are that ICI plus chemotherapy significant changes pathological complete response and tumour regression rates, demonstrating a favourable signal for event free survival, keeping the resection and surgery completion rates comparable and having a manageable safety profile.

The significant improvement in pCR seen in this analysis is consistent with the new understanding that chemotherapy can induce immunogenic cell death and increase tumour antigen presentation, thus providing a different microenvironment that is more favorable to ICI-mediated T-cell activation [13]. This synergistic mechanism has been biologically plausible and has been shown to occur in multiple solid tumour (notaldo) types including non-small cell lung cancer and triple negative breast cancer, in which perioperative chemo-immunotherapy has already transformed clinical practice [14,15]. However, whether the pCR benefit transfers into long-term survival benefit of gastric cancer is an open debate. A recent analysis of surrogate endpoints of pCR in gastroesophageal adenocarcinoma by Peixoto et al. (2026) involving 26 neoadjuvant RCTs concluded that pCR showed weak trial-level correlation with overall survival, implying that the surrogate endpoint should not be blindly taken as a surrogate endpoint [16]. Of note, in KEYNOTE-585, the significantly higher pCR was NOT associated with a statistically significant EFS benefit, but in MATTERHORN, the pCR benefit was associated with improved EFS. These discordant results suggest that factors other than initial pathologic response, such as adequacy of adjuvant ICI continuation and immunological characteristics of residual micrometastatic disease, likely modulate long-term results [17].

The EFS benefit seen in the two trials which have been reporting hazard ratios is very encouraging but must be interpreted with caution given the small number of contributing studies. In contrast, one trial assessing a neoadjuvant ICI component (ATTRACTION-5; that evaluated adjuvant nivolumab after D2 gastrectomy without an adjuvant neoadjuvant component) showed negligible benefit in terms of prolonged RFS [18]. This discrepancy emphasises the possible importance of the perioperative (neoadjuvant plus adjuvant) approach as opposed to the pure adjuvant immunotherapy approach and supports the hypothesis that pre-surgical immune priming against the intact tumour is necessary to maximise the therapeutic benefit of checkpoint inhibition. Similarly, the VESTIGE trial concluded that supporting the recommendation for adjuvant chemotherapy, replacement with nivolumab plus ipilimumab was inferior to continuing periodic chemotherapy, further strengthening the conclusion that ICIs are likely to be most successful when used in combination with, rather than substituted for, standard chemotherapy [19].

The safety in our analysis shown is encouraging in multiple ways. Adverse events in the intervention group were not increased with the addition of ICIs. This is of clinical importance as concerns about perioperative immune-related toxicities delaying surgery or adversely impacting postoperative recovery have been a major barrier to adoption [20]. However, the considerably higher rate of immune related adverse events and discontinuation of therapy because of adverse events warrant careful patient counselling and monitoring. The profile of immune-related adverse events is consistent with known class effects for anti-PD 1 and anti-PD L1 agents and has been found similarly in a meta-analysis by Yang et al. (2024), which report a pooled odds ratio of 4.03 for any grade immune-related adverse events with perioperative ICI combinations [21].

One issue that really needs to be thought about is the role of biomarker-guided patient selection. Subgroup analyses from KEYNOTE-585 and MATTERHORN have suggested that patients with microsatellite instability-high (MSI-H) tumours gain the most benefit from perioperative immunotherapy and the benefit in microsatellite-stable populations, although smaller, also exists [22]. Given that MSI-H tumours only account for about 5-10% of gastric cancers, the best approach for the rest of the microsatellite stable population needs further refinement. The integration of new biomarkers such as PD-L1 combined positive score, tumour mutational burden, and circulating tumour DNA dynamics, may be of use to select patients more precisely in the future [23].

### Limitations

A number of limitations should be recognized. First, only four RCTs met our inclusion criteria and therefore we have limited statistical power, especially for assessing rare events and if an outcome is reported from only two studies. Second, the various trials included were done with different ICI agents (pembrolizumab, nivolumab, atezolizumab, toripalimab/sintilimab), chemotherapy backbones, and dosing schedules, which adds an element of clinical heterogeneity in spite of the consistent direction of effect across trials. Third, survival outcomes (EFS and OS hazard ratios) were only available from two papers, which means that firm conclusions on the long-term survival benefit cannot be drawn. Fourth, we lacked access to individual patient-level data, which was the reason we did not have the opportunity for subgroup analyses by PD-L1 status, MSI status or tumour location. Fifth, the follow-up periods of the trials were varied and still relatively short for strong conclusions on the effect size on overall survival. Finally, publication bias cannot be ruled out due to the small number of included studies.

## Conclusion

This meta-analysis shows that the addition of immune checkpoint inhibitors to perioperative chemotherapy in resectable gastric and gastroesophageal junction cancer has significant benefits in improving pathological complete response and tumour regression rates without any increase in surgical morbidity or adverse events rates overall. The EFS signal has been very promising but needs to be validated with confirmatory data on mature OS. The much higher rate of immune-related adverse events and treatment discontinuation makes close monitoring the need of the hour. For the time being, clinicians should consider the benefits of pathological response against the immune-related toxicity profile on an individualised basis.

## Figures and Tables

**Table 1:** Characteristics of the Included Studies

Variable	Shitara et al. 2024 (ICI+Chemo / Chemo) [7]	Yuan et al. 2024 (ICI+Chemo / Chemo) [10]	Lorenzen et al. 2024 (ICI+Chemo / Chemo) [9]	Janjigian et al. 2025 (ICI+Chemo / Chemo) [8]
**Year**	2024	2024	2024	2025
**Study Design**	Phase 3, DB RCT	Phase 2, OL RCT	Phase II/III, OL RCT	Phase 3, DB RCT
**ICI Agent / Chemo Backbone**	Pembrolizumab / Cis+Cape/5-FU	Toripalimab / SOX or XELOX	Atezolizumab / FLOT	Durvalumab / FLOT
**Median Follow-up**	47.7 months (IQR 38.0–54.8)	NR	NR (phase II)	31.5 momths (IQR 26.7–36.6)
**Total Sample Size**	402 / 402	54 / 54	146 / 149	474 / 474
**Country**	United States, Germany, Spain, Japan, South Korea, United Kingdom, Belgium, Poland and Italy	China	Germany and Switzerland	United States, Germany, Spain, Japan, South Korea, United Kingdom
**Age, median (IQR/range), yr**	64 (56–70) / 63 (55–69)	58 (48–67) / 62 (54–68)	61 (29–79) / 62 (23–80)	62 (26–84) / 63 (28–83)
**Male, n (%)**	288 (72%) / 287 (71%)	35 (64.8%) / 35 (64.8%)	116 (80%) / 101 (68%)	326 (68.8%) / 356 (75.1%)
**Female, n (%)**	114 (28%) / 115 (29%)	19 (35.2%) / 19 (35.2%)	30 (21%) / 48 (32%)	148 (31.2%) / 118 (24.9%)
**ECOG PS 0, n (%)**	302 (75%) / 299 (74%)	36 (66.7%) / 36 (66.7%)	107 (73%) / 114 (77%)	337 (71.1%) / 366 (77.2%)
**ECOG PS 1, n (%)**	100 (25%) / 102 (25%)	18 (33.3%) / 18 (33.3%)	39 (27%) / 35 (24%)	137 (28.9%) / 108 (22.8%)
**Tumor: Stomach, n (%)**	316 (79%) / 322 (80%)	37 (68.5%) / 34 (63.0%)	56 (38%) / 58 (39%)	324 (68.4%) / 316 (66.7%)
**Tumor: GEJ, n (%)**	86 (21%) / 79 (20%)	17 (31.5%) / 20 (37.0%)	90 (62%) / 91 (61%)	150 (31.6%) / 158 (33.3%)
**Lauren: Diffuse, n (%)**	165 (41%) / 183 (46%)	18 (33.3%) / 20 (37.0%)	71 (49%) / 62 (42%)	130 (27.4%) / 119 (25.1%)
**Lauren: Intestinal, n (%)**	192 (48%) / 174 (43%)	36 (66.7%) / 34 (63.0%)	14 (10%) / 16 (11%)	245 (51.7%) / 238 (50.2%)
**cT3, n (%)**	211 (53%) / 219 (54%)	21 (38.9%) / 17 (31.5%)	—	307 (64.8%) / 321 (67.7%)
**cT4/T4a, n (%)**	142 (35%)[T4a] / 145 (36%)[T4a]	33 (61.1%)[cT4a] / 37 (68.5%)[cT4a]	—	117 (24.7%) / 117 (24.7%)
**PD-L1 CPS ≥1 / TAP ≥1%, n (%)**	302 (75%) / 306 (76%)	NR	82 (56%) / 88 (59%)	426 (89.9%)[TAP≥1%] / 427 (90.1%)[TAP≥1%]
**MSI-H / dMMR, n (%)**	104 (26%) / 116 (29%)	3 (5.6%) / 3 (5.6%)	27 (19%) / 26 (17%)	25 (5.3%) / 24 (5.1%)
